# Multi-Response Optimization of Sequential Mild Heating and UV-C Radiation Hurdle Technology for Decontamination and Quality Retention of Traditional Tunisian Coarsely Milled Barley (Chorba Chiir)

**DOI:** 10.3390/foods15213962

**Published:** 2026-11-04

**Authors:** Eya BenSalem, Taroub Bouzaiene, Rania Zayani, Mohamed Boussaid, Carole Chaix, Chokri Messaoud

**Affiliations:** 1 Laboratory of Nanobiotechnologies LR24ES19, Department of Biology, National Institute of Applied Sciences and Technology (INSAT), Carthage University, B.P. 676, Tunis Cedex 1080, Tunisia; zayani.rania@gmail.com (R.Z.); chok.messaoud@yahoo.fr (C.M.); 2 Centre National de la Recherche Scientifique, Institut des Sciences Analytiques, UMR 5280, Université Claude Bernard Lyon 1, 69100 Villeurbanne, France; carole.chaix-bauvais@univ-lyon1.fr; 3 Laboratoire d’Écologie et de Technologie Microbienne, Institut National des Sciences Appliquées et de Technologie (INSAT), Carthage University, B.P. 676, Tunis Cedex 1080, Tunisia; taroub.bouzayane@insat.ucar.tn

**Keywords:** *Hordeum vulgare* L., hurdle technology, Box–Behnken design, heat-resistant spore-forming bacteria, water activity, color kinetics, low-moisture food safety

## Abstract

Low-moisture cereal products, such as traditional Tunisian coarsely milled barley, represent a vital global and regional dietary staple. However, their post-harvest processing is challenged by contamination with highly resilient endogenous spore-forming bacteria. This study investigated a sequential mild-thermal and UV-C hurdle technology, combining oven temperature (Factor A; 60, 80 and 100 °C) thermal treatment time (Factor B; 2, 6 and 10 min) with UV-C radiation (Factor C; 214.8, 644.4 and 1074.0 mJ/cm^2^), using a three-factor, three-level Box–Behnken experimental design coupled with response surface methodology (RSM). Three responses were evaluated: microbial logarithmic reduction (Y1), water activity a_w_ (Y2), and total color difference ΔE (Y3). Statistical analysis confirmed that thermal factors (A: oven temperature and B: thermal treatment time) served as the primary drivers of microbial inactivation, while UV-C light acted primarily at the immediate solid surface, inducing localized photothermal micro-evaporation that significantly affected surface water activity without degrading bulk chromatic properties. Multi-response optimization established an optimal processing window at an oven temperature of 94.5 °C, a thermal treatment time of 8.30 min and, a UV-C dose of 1074 mJ/cm^2^. Experimental validation under these optimal conditions yielded a highly reliable 1.385 ± 0.013 Log CFU/g reduction in naturally occurring, heat-resistant spore-forming bacteria. Concurrently, the product maintained an exceptionally dry state (a_w_ = 0.34 ± 0) and preserved its natural visual attributes (ΔE= 2.77 ± 0.08). The low validation relative standard errors (RSE < 10% for physical attributes; 15.6% for microbial reduction) demonstrated a good predictive precision and industrial feasibility of the quadratic models.

## 1. Introduction

Cereals such as barley (*Hordeum vulgare* L.) are among the most cultivated staple crops globally, ranking fourth in worldwide cereal production after maize, wheat, and rice [1]. Domestically and internationally, barley plays a fundamental role in global food security, serving as a primary source of dietary fiber, minerals, and bioactive compounds [2]. While globally utilized for animal feed, malting, and functional human foods, barley holds particular cultural and dietary significance in North Africa, specifically in Tunisia [2,3]. In Tunisian culinary tradition, coarsely milled barley is an essential ingredient in “Chorba Chiir” (barley soup), a traditional cereal dish consumed regularly as a dietary staple, particularly during the winter season, and commonly purchased by weight from local retail outlets and weekly markets in Tunis [3,4].

Despite its nutritional value, the post-harvest processing and storage of dry barley products present significant microbial safety challenges [5]. Dry particulate matrices are frequently contaminated with desiccation-tolerant vegetative pathogens like *Salmonella enterica* [5,6] and heat-resistant, spore-forming bacteria such as *Bacillus cereus* [5], which persist through conventional drying operations and compromise shelf-life stability. Traditional wet thermal decontamination methods often prove unsuitable for dry cereal products, as elevated moisture and temperatures induce unwanted starch gelatinization, structural degradation, and severe non-enzymatic Maillard browning [7]. Consequently, there is an urgent demand for mild-thermal or non-thermal hurdle strategies capable of achieving microbial reduction while preserving the physical and quality attributes of dry foods. Hurdle technology, which combines thermal and non-thermal processing treatments, offers a promising solution to enhance microbial inactivation synergistically [8,9]. Documented thermal techniques include microwave, radio-frequency, and ohmic heating, whereas non-thermal techniques include pulsed electric field (PEF), ultrasonic, high pressure processing (HPP), cold plasma (CP), ozone, and UV-C irradiation [9,10].

In this context, pairing mild convective oven heating with UV-C radiation presents an effective sequential treatment [11]. While thermal conduction acts as the primary driver for kinetic inactivation within the matrix [12], UV-C light delivers targeted photonic surface decontamination [11]. Physiologically, the mild pre-heating step weakens and increases the fluidity of the bacterial cell membrane [13,14], rendering survivors highly sensitive to subsequent UV-C treatment. UV-C radiation is absorbed by microorganisms, resulting in the formation of DNA photoproducts such as cyclobutene pyrimidine dimers (CPDs) and pyrimidine 6–4 pyrimidone (6–4PP). The structural deformation caused by the formation of these dimers hinders transcription and replication by initiating the formation of new DNA, which causes microorganisms to be inactivated [15,16]. To systematically evaluate and optimize this combined decontamination process, empirical statistical modeling is required. Response surface methodology (RSM) is used for modeling and analysis of a response by evaluating simultaneously several variables, such as heating temperature, UV-C dose, and treatment duration, and the objective is to optimize these responses [17].

Therefore, the objective of this study was to optimize a sequential mild oven heating and UV-C decontamination process for Tunisian coarsely milled barley using response surface methodology. Specifically, this work evaluates the multi-response optimization by maximizing the logarithmic reduction in endogenous bacterial populations (Y1), while minimizing water activity a_w_ (Y2) and total color difference ΔE (Y3) to ensure overall quality preservation.

## 2. Materials and Methods

### 2.1. Food Matrix

**Figure 1:**
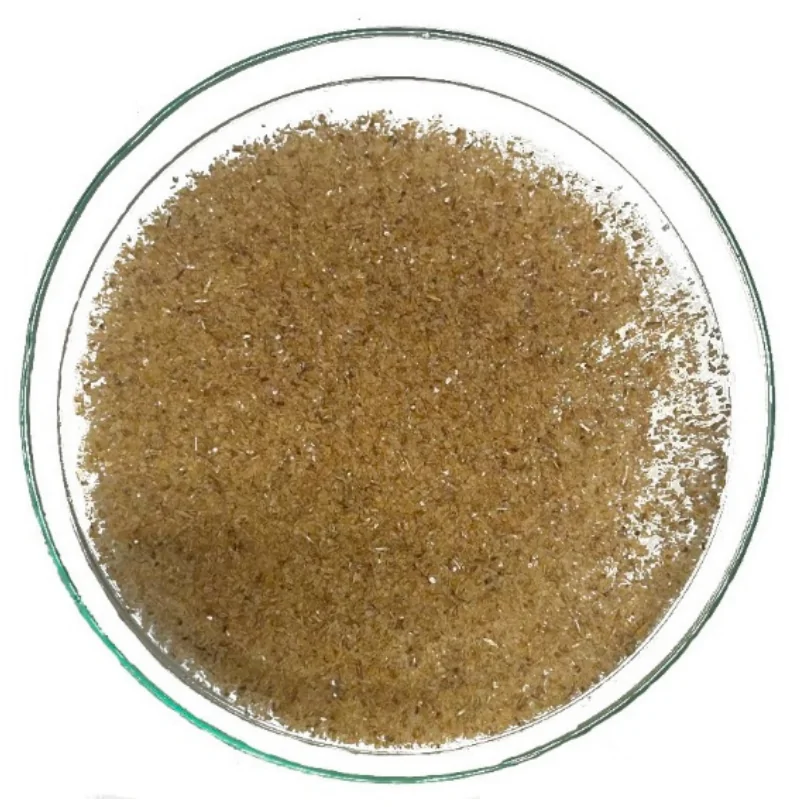
Traditional Tunisian coarsely milled barley (*Hordeum vulgare* L.).

Unpackaged coarsely milled barley (*Hordeum vulgare* L.), was purchased by weight (1 Kg) from local retail outlets (weekly markets in Tunis, Tunisia). The sample (Figure 1) was thoroughly homogenized and stored in a dry place at ambient temperature (25 °C ± 2) until further analysis.

### 2.2. Experimental Design of the Combined Thermal and UV-C Treatment

A response surface methodology (RSM) based on a three-factor, three-level Box–Behnken Design (BBD) was employed to investigate the effects of combined thermal and UV-C treatment on the efficiency of decontaminating coarsely milled barley from endogenous heat-resistant spore-forming bacteria (Log reduction) as well as its impact on key quality parameters such as water activity (a_w_) and total color difference (ΔE). Three independent variables were used (A) oven temperature, (B) thermal treatment time, and (C) UV-C dose. The coded and experimental levels of the independent variables are presented in Table 1. The experimental design consisted of 15 experimental runs, with 12 factorial points and 3 central points.

The experimental ranges of all investigated independent factors (A: oven temperature, B: thermal treatment time, and C: UV-C dose) were selected based on preliminary screening experiments to achieve effective bacterial inactivation while avoiding thermal and photothermal degradation of the sample.

A second-order polynomial regression model was applied to predict each response as a function of the independent variables following Equation (1):

(1) Y = β_0_ + β_A_A + β_B_B + β_C_C + β_AB_AB + β_AC_AC + β_BC_BC + β_AA_A^2^ + β_BB_B^2^ + β_CC_C^2^

where β_0_ is the model intercept; β_A_ β_B_, and β_c_ are the linear coefficients; β_AB_, β_AC_, and β_BC_ are the two-factor interaction coefficients; β_AA_, β_BB_, and β_CC_ are the quadratic coefficients; A, B, and C are the independent variable factors (A: oven temperature, B: thermal treatment time, and C: UV-C dose).

### 2.3. Thermal and UV-C Treatment Decontamination Protocol

The decontamination process for coarsely milled barley was conducted as follows: for each experimental run, a 20 g sample was spread in a thin, uniform layer inside a sterile glass Petri dish (180 mm diameter). The layer thickness was measured to be 1.2 mm ± 0.3. Particle size distribution was determined using an Octagon 200 sieve shaker (Endecotts Ltd., London, UK) equipped with a stack of standard analytical sieves (Endecotts Ltd., London, UK) (ranging from 0.075 to 2.0 mm), revealing a final particle size ranging from 0.5 mm (500 µm) to 1.5 mm.

Thermal treatments were carried out using a forced-convection laboratory oven (BINDER GmbH, Tuttlingen, Germany) preheated to the temperatures (A: 60, 80, or 100 °C). To monitor temperature stability directly, a calibrated precision mercury-in-glass thermometer (±0.5 °C accuracy) was placed inside the oven chamber. Once the oven chamber reached and stabilized at the target temperature, samples were inserted and held for the total designated thermal treatment time (B: 2, 6, or 10 min). It is recognized as a methodological limitation that the recorded temperature corresponds to the oven chamber air rather than the direct core or surface temperature of the barley samples. However, given the thin sample layer (1.2 ± 0.3 mm), rapid heat conduction across the coarsely milled barley particles was assumed to reach rapid thermal equilibrium during the total residence holding time. UV-C irradiation was performed using a dual-tube UV lamp system (Model VL-215.LC, Vilber Lourmat, Collégien, France; 30W total power rating) containing a 15W germicidal tube emitting predominantly at λ = 254 nm. To ensure safety and exclude environmental light interference, both the UV lamp and the sample dish were placed inside a custom opaque enclosure. The UV-C lamp was turned on 15 min prior to the treatment to ensure light stability. The sample dish was positioned at a fixed vertical distance of 10 cm directly beneath the UV-C tube, providing a nominal UV-C irradiance (I) of 3.58 mW/cm^2^ at the sample surface level, which was verified using a calibrated digital UV-C radiometer (Vilber Lourmat, France). Exposure durations of 1 min (60 s), 3 min (180 s), and 5 min (300 s) were selected based on preliminary screening trials and corresponded to UV-C doses of 214.8, 644.4, and 1074.0 mJ/cm^2^, respectively.

The applied UV-C dose (mJ/cm^2^) was calculated using Equation (2):

(2) D = I × t

where I is the UV-C irradiance (mW/cm^2^) and t is the exposure time (s).

To ensure uniform photon distribution and minimize particle self-shadowing, a standardized manual agitation was performed under aseptic conditions at the midpoint of each UV-C exposure (30 s, 1 min 30 s, and 2 min 30 s). The Petri dish containing the thin sample layer (1.2 ± 0.3 mm) was gently shaken and mixed with a sterile spatula for 5 s, following recommended strategies for particulate food decontamination [11]. Due to the limited penetration depth of UV-C light in opaque solid tissues, particle self-shielding remains an inherent physical limitation, and the reported nominal dose reflects surface-applied fluence rather than a volumetric dose.

For every experimental run, the sample dish was positioned in the middle of the oven chamber and in the middle of the enclosed UV-C chamber. Both treatments were performed sequentially, starting with the thermal treatment followed by UV-C treatment. A transfer delay of 10s was recorded between the treatments, corresponding to the manual removal of the sample dish from the oven chamber and its immediate transfer under the UV-C lamp. An untreated coarsely milled barley sample served as the control (25 °C (room temperature), 0 min, 0 mJ/cm^2^).

### 2.4. Analytical Experiments

#### 2.4.1. Microbiological Analysis and Enumeration

Microbiological analysis was performed to quantify the survival of endogenous heat-resistant spore-forming bacteria following the decontamination process. A 10 g sample of treated or untreated (control) coarsely milled barley sample was added to 90 mL of sterile saline solution (0.9% NaCl) in an Erlenmeyer flask and homogenized for 1 min by vortex. This suspension consisted of the first dilution (d = 10^−1^). A heat-shock step was performed at 80 °C for 10 min in a controlled water bath (BIOBASE, Jinan, China) to eliminate vegetative cells [18]. The suspension was cooled down, and decimal serial dilutions were prepared in sterile saline. 0.1 mL were spread plated onto Tryptic Soy Agar plates (BIOKAR Diagnostics, Beauvais, France, BK047HA)in triplicate. The Petri dishes were incubated aerobically at 30 °C for 24 h. After incubation, colonies were counted, and the results are expressed as Log colony-forming units per gram (log CFU/g). The decontamination efficacy was evaluated as Log reduction (Y1) following Equation (3):

(3) Log Reduction (Log CFU/g) = log_10_N_0_ − log_10_N_t_

where N_0_ is the initial count of the untreated sample (CFU/g) and N_t_ is the residual count after treatment (CFU/g).

#### 2.4.2. Water Activity (a_W_) Determination

Water activity a_W_ (Y2) of treated and untreated (control) coarsely milled barley samples was measured in triplicate at 25 °C ± 2 using an electrolytic water activity meter (LabTouch-aW, Novasina AG, Lachen, Switzerland). Prior to measurements, the instrument was calibrated as described by the manufacturer and readings were noted when equilibrium was reached.

#### 2.4.3. Total Color Difference (ΔE) Determination

Color parameters of untreated and treated coarsely milled barley samples were measured in triplicate using a handheld portable colorimeter (Linshang Technology Co., Ltd., Shenzhen, China) calibrated beforehand following the manufacturer’s guidelines. Color was recorded in the CIELAB system through the parameters L^*^, a^*^, and b^*^. The total color difference ΔE (Y3) between treated and untreated samples was calculated using the following equation:

(4) $$\Delta E=\sqrt{{\left(L^{*}-L_{0}^{*}\right)}^{2}+{\left(a^{*}-a_{0}^{*}\right)}^{2}+{\left(b^{*}-b_{0}^{*}\right)}^{2}}$$

where L_0_^*^, a_0_^*^, and b_0_^*^ are the color coordinates of the untreated (control) sample, and L^*^, a^*^, and b^*^ are the color coordinates of the treated samples.

### 2.5. Statistical Analysis

The experimental measurements were performed in triplicate (*n* *= 3*) and are presented as mean ± standard deviation (SD). To evaluate significant differences between the 15 Box–Behnken experimental runs for each response (Y1: Log reduction, Y2: water activity a_w_, Y3: total color difference ΔE), one-way analysis of variance (ANOVA) was conducted using IBM SPSS statistics (Version 20), followed by Duncan’s multiple range test for post hoc comparisons at *p* < 0.05. Response surface methodology (RSM), second-order polynomial regression models, coefficient of determination (R^2^, adj R^2^), evaluation of linear, quadratic, and interaction factor effects, construction of 3D response surface plots, and multi-response optimization via the Derringer desirability function (D) were carried out using Statgraphics Centurion software (Version 19), with *p* < 0.05. To assess the predictive reliability of the optimized mathematical models, experimental validation was performed in triplicate under the predicted optimal processing conditions, and the resulting experimental values were compared against the model-predicted response values (RSE %).

## 3. Results

### 3.1. Box–Behnken Experimental Matrix and Observed Responses

The experimental design matrix and the observed response values for the 15 Box–Behnken runs are summarized in Table 2. Three responses were evaluated on the coarsely milled barley samples: microbial logarithmic reduction (Y_1_), water activity a_w_ (Y_2_), and Total Color Difference ΔE (Y_3_). Across all 15 experimental modalities, significant differences (*p* < 0.05) were observed for all three evaluated responses. Following combined processing, microbial logarithmic reduction (Y_1_) ranged from 0.468 ± 0.068 Log CFU/g (Run 1: 60 °C, 2 min, 644.4 mJ/cm^2^) to a maximum of 1.532 ± 0.056 Log CFU/g (Run 4: 100 °C, 10 min, 644.4 mJ/cm^2^). Residual water activity (Y_2_) decreased across all treatments, maintaining a narrow range between 0.34 and 0.36. Total color difference ΔE (Y_3_) values ranged from 3.09 ± 0.076 (Run 8: 100 °C, 6 min, 1074 mJ/cm^2^) to 9.82 ± 0.083 (Run 3: 60 °C, 10 min, 644.4 mJ/cm^2^).

**Figure 2:**
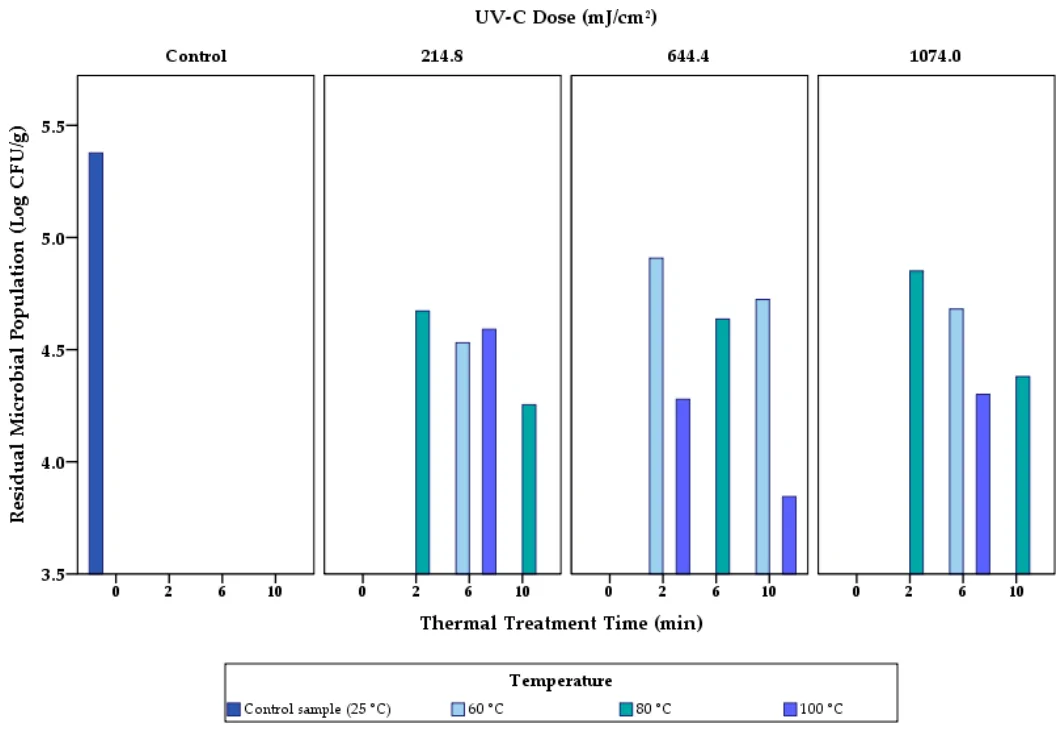
Impact of combined thermal–UV-C hurdle processing on residual microbial loads.

The untreated coarsely milled barley control sample (25 °C, 0 min, 0 mJ/cm^2^) yielded an initial spore-forming microbial load of 5.377 Log CFU/g (Figure 2). Across all Box–Behnken experimental modalities, a distinct temperature-dominated inactivation trend was observed. Extending thermal treatment time from 2 to 10 min at higher oven temperatures (80 and 100 °C) resulted in a significant decline in the microbial population relative to the untreated sample. However, the increase in UV-C doses from 214.8 to 1074.0 mJ/cm^2^ across constant oven temperatures resulted in minimal variation in residual microbial population counts.

### 3.2. Statistical Analysis and ANOVA of Response Models

To evaluate the individual and combined effects of the factors, analysis of variance (ANOVA) was performed for linear, interaction, and quadratic terms across all responses, as detailed in Table 3. For microbial logarithmic reduction (Y1), thermal processing parameters exercised a dominant effect on heat-resistant spore-forming bacteria. Both oven temperature (Factor A, *p* = 0.0497) and thermal treatment time (Factor B, *p* = 0.0486) were statistically significant. In contrast, UV-C dose (Factor C, *p* = 0.7888), two-factor interactions (AB, AC, BC), and quadratic terms (AA, BB, CC) showed no significant impact (*p* > 0.05). As for water activity (Y2), UV-C dose exhibited a statistically significant main effect (Factor C, *p* = 0.0409). Additionally, the oven temperature and thermal treatment time interaction (AB, *p* = 0.0493) and the quadratic oven temperature term (AA, *p* = 0.0268) significantly governed water activity shifts. None of the linear factors (A, B, C), interaction terms (AB, AC, BC), or quadratic terms (AA, BB, CC) significantly influenced total color difference (Y_3_, *p* > 0.05).

### 3.3. RSM Equations and 3D Plots Analysis

Response surface methodology (RSM) was used to evaluate the optimal experimental conditions to maximize (Y1) and minimize (Y2) and (Y3) by establishing the mathematical relationship between the independent variables A: oven temperature, B: thermal treatment time and C: UV-C dose and the response variables. Consequently, second-order polynomial equations for (Y1) microbial Log reduction, (Y2) water activity a_w_, and (Y3) total color difference ΔE were generated by the software Statgraphics 19 and are presented in Table 4.

The model equation for water activity (Y2) yielded the highest coefficient of determination (R^2^ = 86.607%), followed by logarithmic reduction (Y1, R^2^ = 79.927%). Total color difference ΔE (Y3) demonstrated a lower coefficient of determination (R^2^ = 65.320%) and a non-significant overall model effect (*p* = 0.5084, Table 4), suggesting that color variation among samples was less dependent on linear or quadratic terms of the studied experimental variables within the selected domain. To further evaluate model adequacy and statistical validity, diagnostic residual plots for the three evaluated responses, including the normal probability plot and the residuals vs. predicted plot, are provided in the Supplementary Materials (Figures S1–S3). These plots confirm error normality and constant variance across the experimental domain. Individual single-response optimal values and experimental conditions are summarized in Table S1.

To assess the interaction effects of the factors on the evaluated responses, Y1, Y2, and Y3 3D response surface plots were generated based on model equations (Table 4). The 3D plots show the interaction between A: oven temperature; B: thermal treatment time across low (214.8 mJ/cm^2^), intermediate (644.4 mJ/cm^2^), and high (1074.0 mJ/cm^2^) levels of factor; and C: UV-C dose.

**Figure 3:**
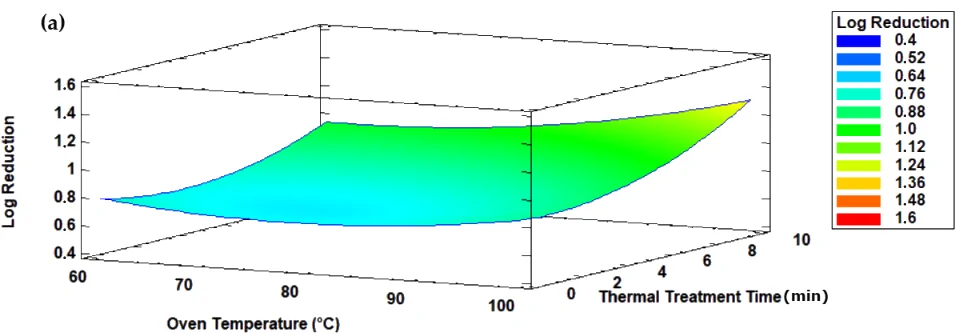

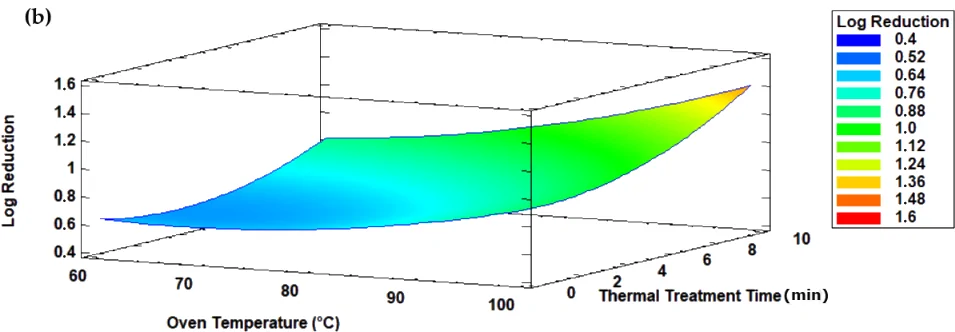

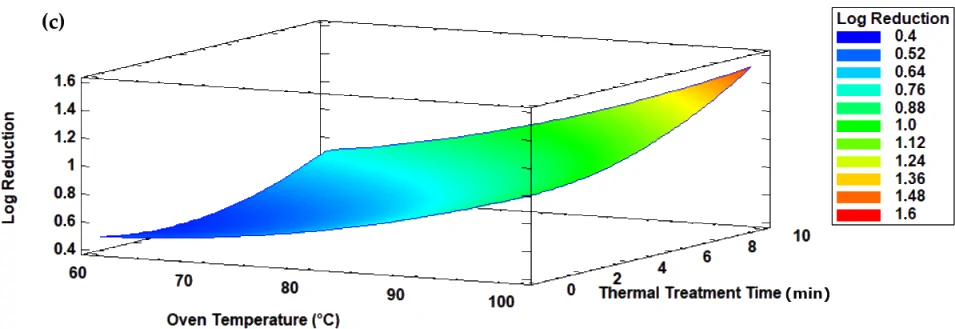
Three-dimensional response surface plots of microbial log reduction (Y1). (**a**) UV-C dose 214.8 mJ/cm^2^, (**b**) UV-C dose 644.4 mJ/cm^2^, and (**c**) UV-C dose 1074.0 mJ/cm^2^.

Response surface plots of Y1 demonstrate a strong temperature–time interaction at elevated thermal levels (Figure 3a–c). At lower temperature intervals [60 °C–70 °C], extending the thermal treatment time from 2 to 10 min results in a minor increase in microbial inactivation. Conversely, at higher oven temperatures (100 °C), the time of thermal exposure demonstrates a stronger non-linear impact on Y1. The maximum microbial Log reduction (>Log 1.4 CFU/g) is reached with combined high oven temperature and thermal treatment time (100 °C and 10 min). A UV-C dose increase from 214.8 to 1074.0 mJ/cm^2^ shifts the surface slightly upward, generating higher Y1 responses.

**Figure 4:**
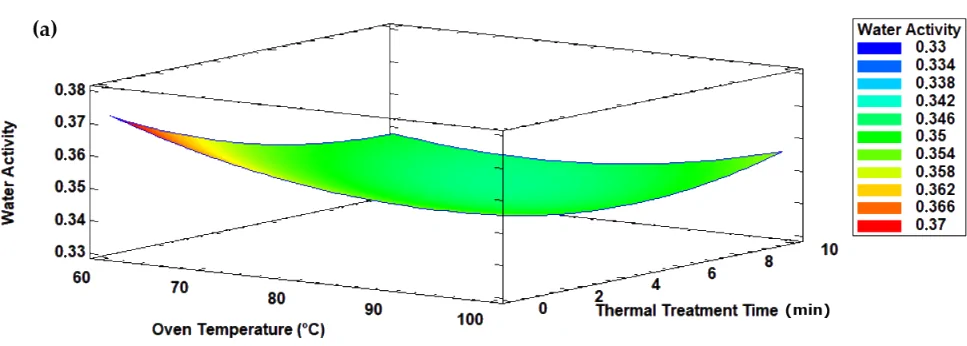

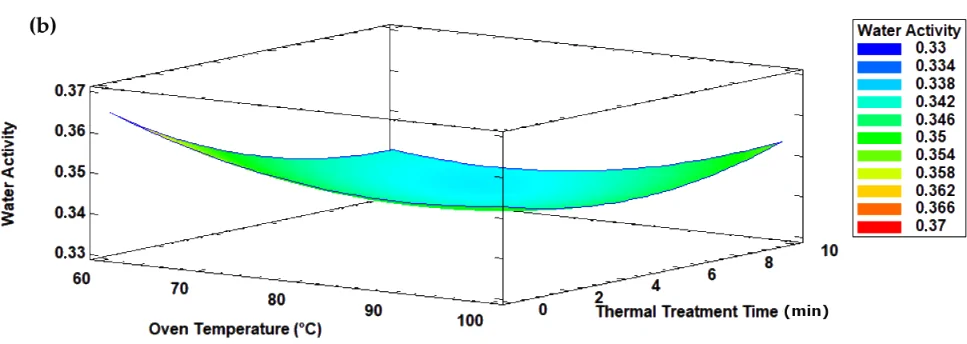

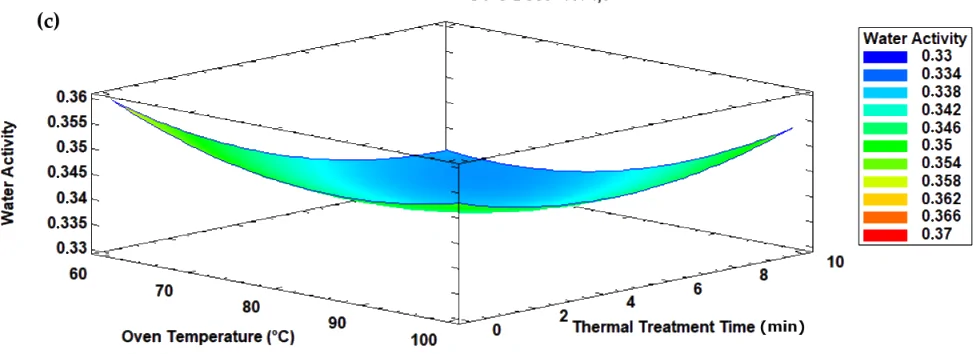
Three-dimensional response surface plots of water activity a_w_ (Y2). (**a**) UV-C dose 214.8 mJ/cm^2^, (**b**) UV-C dose 644.4 mJ/cm^2^, and (**c**) UV-C dose 1074.0 mJ/cm^2^.

All 3D plots for Y2 (Figure 4a–c) show a concave shape occurring at the intermediate oven temperature interval [75–85 °C], due to the significant quadratic effect AA (*p* = 0.0268, Table 3) and water activity values range from 0.33 to 0.34. A combination of low oven temperature (60 °C) and short thermal treatment time (2 min) retains higher water activity (0.36–0.37), whereas processing at 100 °C for 10 min maintains a_w_ below 0.35.

**Figure 5:**
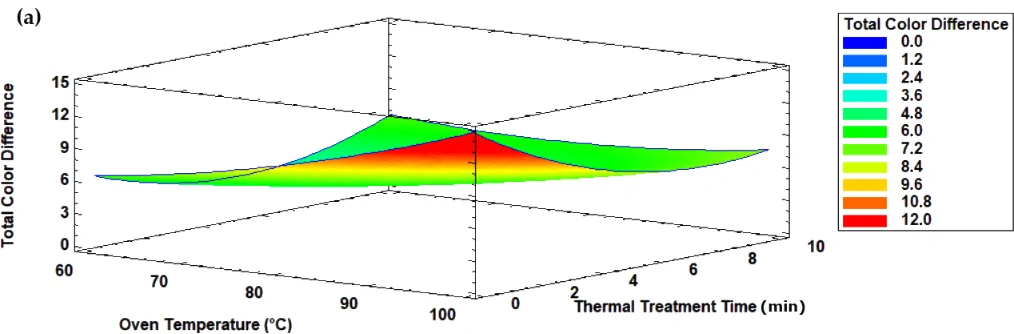

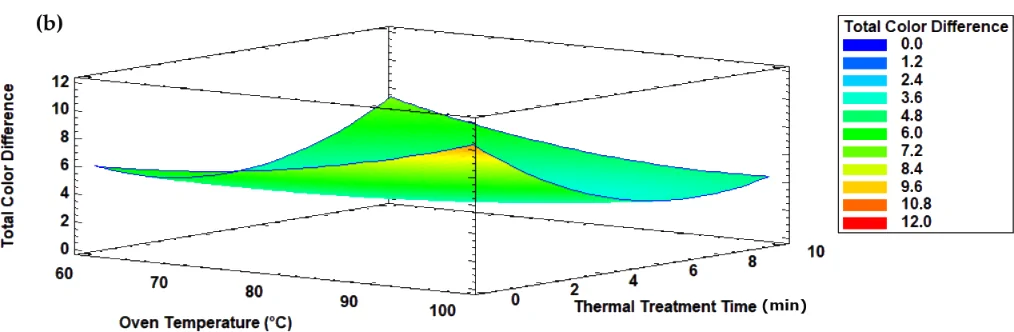

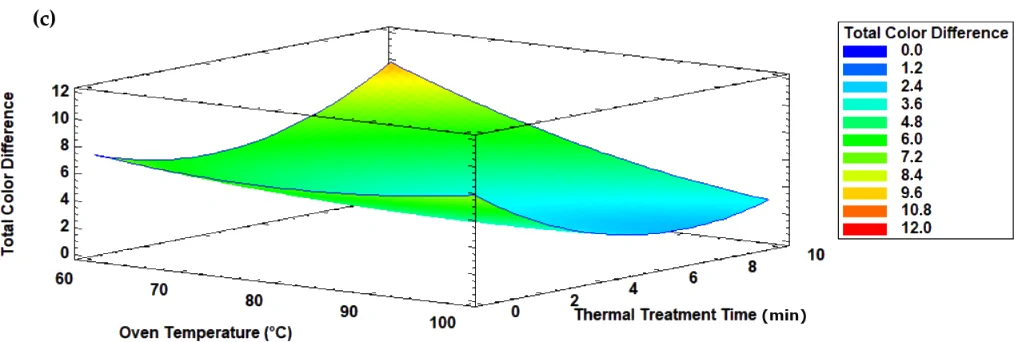
Three-dimensional response surface plots of total color difference ΔE (Y3). (**a**) UV-C dose 214.8 mJ/cm^2^, (**b**) UV-C dose 644.4 mJ/cm^2^, and (**c**) UV-C dose 1074.0 mJ/cm^2^.

Response surface plots of Y3 (Figure 5a–c) exhibit an almost flat topology across most factor combinations, confirming the non-significant linear, quadratic, and interaction effects of these factors (*p* > 0.05, Table 3). A slight increase in ΔE values at high oven temperature (100 °C) and low thermal treatment time [0–2 min] under low (214.8 mJ/cm^2^) and moderate (644.4 mJ/cm^2^) UV-C doses is observed. However, combined low oven temperature (60 °C), extended thermal treatment time (10 min) and high UV-C dose (1074.0 mJ/cm^2^) shows high ΔE values reaching 10 (Figure 5c).

### 3.4. Multi-Response Optimization and Model Validation

To determine the optimal processing window that simultaneously maximizes microbial logarithmic reduction (Y1) and minimizes water activity (Y2) and total color difference (Y3), a multi-response optimization was performed using Derringer’s desirability function (D). The resulting optimal condition combination (oven temperature of 94.5 °C, thermal treatment duration of 8.30 min, and UV-C dose of 1074.0 mJ/cm^2^), alongside the model-predicted values, actual experimental validation results, and relative standard rrrors (RSE %), are presented in Table 5.

The comparison between the predicted values and the actual experimental values was assessed using the relative standard error (RSE %). The predicted microbial logarithmic reduction (Y1) response of 1.198 Log CFU/g was lower than the experimental value of Log 1.385 CFU/g (RSE = 15.6%). For water activity aw (Y2), the model demonstrated excellent predictive precision with an RSE value of 0%. However, for total color difference ΔE (Y3), the model predicted ΔE value of 2.60, whereas the experimental validation yielded ΔE value of 2.77 with an RSE value of 6.53%.

## 4. Discussion

The decontamination of cereal-based foods presents a distinct technological challenge since the preservation of initial matrix quality is essential and often restricts the efficiency of the decontamination process. To address this constraint, the present study evaluated the application of combined mild oven heating with temperatures ranging from 60 to 100 °C under short thermal treatment time (2, 6, and 10 min) and the application of UV-C radiation (214.8, 644.4, and 1074.0 mJ/cm^2^). The samples were subjected to sequential decontamination process starting with thermal treatment followed by UV-C irradiation aligning with the findings of Gayán et al., suggesting that combined UV-C and heat treatment used as hurdle technology demonstrated higher *Salmonella enterica* [19] and *Escherichia coli* [13] inactivation kinetics. By employing a Box–Behnken experimental design coupled with response surface methodology (RSM), the quadratic models provided comprehensive insights into the primary factors governing microbial logarithmic reduction (Y1), water activity a_w_ (Y2), and total color difference ΔE (Y3). Multi-response optimization successfully identified processing windows that maximize heat-resistant spore-forming bacterial inactivation while preserving the essential physical properties of coarsely milled barley. In the present study, the sequential thermal and non-thermal treatment yielded a maximum microbial reduction of 1.532 Log CFU/g, when applying an oven temperature of 100 °C and thermal treatment time for 10 min, starting from an initial load of 5.377 Log CFU/g. In dry cereal matrices, total sterilization is technically unfeasible without compromising grain quality. According to ICMSF [20] safety guidelines for raw cereal ingredients, an aerobic spore count below 10^4^ CFU/g (4 Log CFU/g) is commercially safe. Furthermore, maintaining a_w_ between 0.34 and 0.36 well below the germination limit (a_w_ < 0.92) ensures surviving spores remain physiologically dormant during ambient storage. The quadratic response model (Y1) demonstrated that A: oven temperature and B: thermal treatment time were the factors that exercised a dominant and statistically significant effect (*p* < 0.05) on heat-resistant spore-forming bacteria inactivation, whereas UV-C irradiation doses (Factor C) did not significantly affect response Y1 (*p* > 0.05). To our knowledge, previous studies were usually conducted on inoculated bacteria in the tested sample and do not assess the effect of hurdle technology on natural microbial contamination. However, Xu et al. [21] evaluated the effectiveness of radio frequency (RF) heating as a physical pasteurization method for buckwheat kernels focusing on natural microbial population and inoculated pathogens such as *Salmonella Typhimurium*, *Escherichia coli*, *Cronobacter sakazakii*, and *Bacillus cereus*. The initial microbial counts for the different collected buckwheat samples ranged from 3.4 to 6.2 Log CFU/g and the application of an RF treatment at 90 °C and 0 min holding time (90 °C-0min) resulted in the highest reduction by 2.8 Log CFU/g for total bacteria. The authors also note that the natural microbial population was more resistant than inoculated pathogens. The microbial reduction in inoculated pathogens, initially at 10^8^ CFU/g, reached 4 Log CFU/g at the same applied treatment (90 °C-0min). This finding highlights that laboratory inoculated pathogens may not always fully represent the challenges of naturally contaminated grains that could have been exposed to dry environmental stress for longer periods, conferring them a higher resistance and tolerance to extra stress conditions such as the RF heating treatment. Building on these findings, Xu et al. [22] discussed how combined hurdle treatments can mitigate tailing phenomena observed during single-method decontamination. However, because our response surface methodology did not include continuous time-course kinetic curves or direct surface imaging, physical protective mechanisms in our barley matrix are presented as plausible hypotheses rather than empirically proven tailing phenomena (Cerf [23]). These protective mechanisms differ fundamentally by hurdle: optical self-shadowing, surface roughness, and non-penetration into opaque grain crevices primarily limit UV-C efficacy. Conversely, thermal resistance is driven by low water activity (a_w_ ≤ 0.36), which stabilizes spore core structures against heat denaturation alongside conductive heat transfer resistance within the solid grain matrix. In different previous studies, even though the combination of UV-C irradiation with other thermal treatments showed a synergistic effect on the microbial population inactivation [13,14,24,25,26], our study demonstrated that UV-C Doses (Factor C) had a non-significant effect (*p* > 0.05) compared to thermal factors namely oven temperature (A) and thermal treatment time (B). This observation could be explained by the physical and optical properties of the cereal matrix. Coarsely milled barley possesses a highly irregular, opaque surface structure containing micro-crevices and overlapping pericarp structures. These irregularities could cause shadowing and shielding adherent bacteria on the surface from direct UV-C photon penetration [27,28,29]. Although a manual agitation step was introduced at mid-point of the UV-C exposure to minimize self-shadowing and ensure a monolayer distribution for the barley samples in our study, 254 nm photons possess a limited penetration depth and act at the immediate surface [30,31]. This phenomenon could explain the dominance of thermal treatment towards decontaminating the samples observed in our study by transferring heat energy into the inner pericarp of barley, disrupting cell membranes of the microbial population, while UV-C provided localized surface decontamination.

Water activity (a_w_) is a fundamental thermodynamic parameter and is defined as the ratio of water vapor pressure to the saturation vapor pressure of pure water at the same temperature [5,12,32]. Unlike total moisture content, water activity represents the chemical potential, free energy and escaping tendency of water molecules [33,34]. Maintaining a water activity value below the critical threshold value of 0.60, ensures physical integrity, biochemical stability and long-term storage and shelf life periods for low moisture food matrix characterized by a_w_ values < 0.85 such as cereals [12,35]. In the present study, untreated coarsely milled barley exhibited an initial water activity of a_w_ = 0.40. Following the thermal and UV-C treatment across the Box–Behnken experimental matrix, the a_w_ of the treated samples showed a significant decrease from untreated sample with a_w_ values ranging from 0.34 to 0.36. When evaluating the factors’ main effect on the response (Y2), only Factor C (UV-C dose) demonstrated a statistically significant effect (Factor C, *p* = 0.0409), while oven temperature (Factor A, *p* = 0.4032) and thermal treatment time (Factor B, *p* = 0.1275) were non-significant (*p* > 0.05). Despite the statistical significance of Factor C, the overall variation in a_w_ across the applied UV-C doses (214.8, 644.4, and 1074.0 mJ/cm^2^) remained narrow, ranging from 0.34 to 0.36. These findings are consistent with the known non-thermal, surface-limited behavior of UV-C photons [11]. As previously mentioned, UV-C light has negligible penetrative depth in solid, opaque food matrices; its germicidal effect is highly restricted on solid surfaces such as almonds [22] or black peppercorns [36]. This limitation is primarily due to the physical topography of solid food particles, where crevices, cavities, and pores cause a severe shadowing effect that protects bacteria from direct radiation [11,22]. Apart from shadowing and shielding effect of solid food matrix, as reported in the previous literature of Xu et al. and Xie et al. [22,36], continuous UV-C exposures causes localized surface heating reaching up to 65 °C that induces thermal micro-evaporation rather than driving bulk moisture desorption. Although this photo thermal mechanism was not directly measured in the present study, it provides a plausible explanation for the observed water activity shifts.

In contrast, the two-factor interactions’ effect in this study revealed that only Oven temperature and thermal treatment time interaction (AB) was significant (AB interaction, *p* = 0.0493) even though the two factors’ main effect was assessed as non-significant (*p* > 0.05). This observation highlights that moisture loss in the treated samples could be driven by a coupled time–temperature thermodynamic mechanism. As discussed by Tadapaneni et al. and Jin et al. [33,34], elevated thermal processing temperatures dynamically shift water activity by disrupting the hydrogen bonds holding water molecules within starch and protein rich matrix of low-moisture foods. Conversely, hydrophobic lipid-rich matrices show minimal a_w_ shifts with temperature because fat molecules do not form hydrogen bonds with water, leaving more free water in the system [12,34]. However, water transport in dense solids is a transient diffusion process, requiring adequate exposure time to physically equilibrate and relocate moisture out of the grain structure, a mechanism supported by the diffusion models of Syamaladevi et al. [37].

The final evaluated response in this study was total color difference ΔE (Y3). From a sensory standpoint, a ΔE value greater than 3.5 is considered as perceptible to unexperienced human eye in the CIELAB color system [38]. In this current study, the ΔE values were calculated between untreated and treated barley samples and ranged from 3.09 ± 0.076 to 9.82 ± 0.083. The different experimental runs of the Box–Behnken matrix showed statistically significant differences (*p* < 0.05, Table 2) implying that the thermal and UV-C treatment induced a noticeable color shift compared to the raw barley control. However, the analysis of individual main effects (Table 3) demonstrated that the total color difference was not significantly governed by any of the factors (*p* > 0.05). Similarly, the factors’ interaction effect showed non-significant interactive effects (*p* > 0.05) for all the combinations (AB, *p* = 0.2499; AC, *p* = 0.1384; BC, *p* = 0.6996; Table 3).

Regarding the impact of thermal treatment, color change in starchy grains during processing is attributed to non-enzymatic Maillard browning reactions and pigment degradation. As Schoeman and Manley [39] demonstrated for wheat grains, dry convective heating within moderate temperature windows preserves natural grain pigments and prevents moisture-activated browning. This observation is supported also by Bai et al. and Dang et al. [40,41], who observed that whole-grain barley and highland barley flour exhibit high intrinsic color stability under controlled dry thermal processing. Mechanistically, as discussed by Syamaladevi et al. and Tadapaneni et al. [12,33], Maillard browning is highly dependent on water activity, typically reaching its maximum velocity at intermediate ranges (a_w_ = 0.65–0.75). Since the applied process in this study successfully reduced and maintained the coarsely milled barley sample’s water activity within an extremely dry state (a_w_ = 0.34–0.36), the high local viscosity of the starch matrix restricted the molecular mobility of reducing sugars. This glassy state kinetically suppressed the Maillard cascade, preventing severe heat-induced darkening and explaining why Factor A (oven temperature) and Factor B (thermal treatment time) failed to exhibit statistically significant main effects.

As for the light-induced surface reaction, the non-significance of the UV-C dose (Factor C) is highly consistent with the known non-thermal, surface-bound behavior of ultraviolet light. Due to its lack of penetrative depth in solid, opaque, and geometrically irregular particulates, UV-C photons cannot penetrate past the outer pericarp layers, protecting the bulk of the barley grain from photo-degradation [11]. Kömürlü et al. [42] further supported this UV-C surface effect, who found that germicidal UV-C treatments of whole wheat and white bread did not significantly affect CIELAB color parameters, preserving high visual uniformity. Similarly, Demir and Elgün [43] reported that stabilizing wheat flour branny fractions with UV-C did not negatively influence color characteristics. While Xie et al. [36] showed that continuous UV-C exposure on solid particulates can cause localized photothermal heating and potential surface discoloration.

From an industrial and technological perspective, this lack of statistical sensitivity in the overall total color difference ΔE response (Y3) represents an exceptional process robustness. In commercial, large-scale processing, operating parameters such as oven temperature gradients or UV-C lamp output frequently fluctuate. The fact that the samples maintain a stable, uniform color across all tested levels (60 to 100 °C, 2 to 10 min, and 214.8 to 1074 mJ/cm^2^) demonstrates that the combined hurdle system achieved a robust operating window. It effectively decontaminates the grain surface without risking the severe localized burning, starch gelatinization, or structural collapse commonly associated with high-humidity steam or wet thermal pasteurization.

The aim of this study was to maximize the logarithmic microbial reduction (Y1) of heat-resistant spore-forming bacteria while minimizing water activity (Y2) and total color difference (Y3) of the coarsely milled barley samples. To identify the optimal operational conditions for the combined thermal and UV-C treatment, response surface methodology (RSM) was employed, and the quadratic models were optimized.

Regarding the logarithmic microbial reduction (Y1, R^2^ = 79.94), the response surface plots revealed a strong temperature-time interaction. This represents a classic thermal effect where heat weakens the bacterial cell membrane, increases its fluidity, and makes the bacterial cells more sensitive to subsequent UV-C exposure, as demonstrated by Gayan et al. and Cheon et al. [13,14].

For the quadratic model of water activity (Y2, R^2^ = 86.607%), the observed concave curvature in the 3D plots are driven by the significant quadratic temperature term (A^2^, *p* = 0.0268), suggests that water desorption kinetics are driven by intermediate temperature ranges [75–85 °C]. Under low oven temperature values (60 °C) and minimal thermal treatment duration (0–2min), the significant interaction AB (*p* = 0.0493) restricted moisture release and maintained the barley samples at higher a_w_ values (0.36–0.37). This restriction was analyzed by Jin et al. [34], as starch–water hydrogen bonds remain strong, whereas increasing thermal parameters breaks these bonds, releasing free water molecules to the surface of carbohydrate-rich matrix as suggested by Tadapaneni et al. [33]. The application of higher UV-C doses up to 1074.0 mJ/cm^2^ lowered the response surface curvature (a_w_ values below 0.35) which may be caused by small photo-thermal evaporation at the outer layer of the barley samples following the observation of Xie and Hung [36].

The quadratic model for total color difference ΔE (Y3) presented a lower fit (R^2^ = 65.320%), indicating that color changes in the samples are governed by a complex multifactorial phenomenon that is less dependent on linear and quadratic variables within the studied domain. The 3D surface plots were characterized by a relatively flat shape at low and moderate UV-C doses (214.8 and 644.4 mJ/cm^2^), with ΔE values ranging from 4.8 to 7.2. This color preservation pattern could be due to low-moisture values blocking Maillard browning pathways [39].

In commercial food processing industries, optimizing individual quality or safety attributes separately is hardly feasible due to different operational conditions. On the one hand, high temperatures and extended thermal treatment durations would maximize microbiological safety; they could negatively impact consumer-accepted quality attributes by accelerating thermal browning and photo-chemical degradation. On the other hand, mild temperatures and shorter treatment durations that preserve color could fail to achieve the required decontamination standards. Regarding statistical model adequacy and optimization reliability, the significant lack-of-fit observed for Y1 (*p* = 0.0002) and Y3 (*p* = 0.0000) is attributable to the extremely low pure error variance among center-point replicates (high experimental repeatability) rather than inadequate polynomial fitting. Although Y3 exhibited a non-significant overall model (*p* = 0.5084), the flat response surface plots reflects process robustness, indicating that decontamination can be achieved without inducing significant color degradation across the operating window. Therefore, multi-response optimization was conducted using Derringer’s desirability function [44]. The optimized experimental conditions were set at 94.5 °C (Factor A), 8.30 min (Factor B), and 1074.0 mJ/cm^2^ (Factor C) with an overall desirability value of D = 0.76. To confirm the accuracy of the predicted model, the experimental values were compared against the model’s predictions using the relative standard error (RSE %) [45]. The experimental logarithmic microbial reduction (Y1) reached 1.385 ± 0.013 Log CFU/g against a predicted value of 1.198 Log CFU/g, yielding an RSE of 15.6%. Water activity (Y2) was experimentally measured at 0.34 ± 0 (predicted value: 0.34) with a perfect RSE of 0%, while the total color difference ΔE (Y3) was evaluated at 2.77 ± 0.08 (predicted value: 2.60), resulting in an RSE of 6.53%. According to Nayak et al., Tran et al., and Lestari et al. [45,46,47], RSE values below 10% represent outstanding predictive precision [45,46,47]. RSE values below 20% are highly acceptable for biological systems, where minor natural variations in bacterial cell stress resistance, initial physiological states, and surface-adhesion topography on irregular grain surfaces introduce inherent experimental noise [12,22,36].

Ultimately, these low RSE values suggest that the developed quadratic polynomial models could be used as commercially viable tools for designing, predicting, and scaling combined thermal–UV-C hurdle decontamination systems for cereal-based matrices.

## 5. Conclusions, Limitations, and Perspectives

This study demonstrates the efficacy of a sequential combination of oven heating and UV-C radiation as a mild thermal and UV-C hurdle strategy for the decontamination of traditional Tunisian coarsely milled barley. The multi-response optimization successfully established an efficient processing window at an oven temperature of 94.5 °C, a thermal treatment time of 8.30 min, and a UV-C dose of 1074 mJ/cm^2^. Under these validated conditions, the combined hurdle system achieved a validated 1.385 ± 0.013 Log CFU/g reduction in naturally occurring heat-resistant spore-forming bacteria without compromising quality attributes, namely water activity (a_w_) and total color difference ΔE. Optimization via response surface methodology confirmed that thermal energy serves as the primary driver of microbial inactivation, while UV-C radiation provides surface-level decontamination. A primary physical limitation of this study lies in the indirect monitoring of sample temperature via the oven chamber atmosphere. Additionally, the application remains limited by the penetration depth of UV-C photons. To overcome these physical limitations and facilitate commercial adoption of this process, future research should focus on incorporating real-time infrared thermography or fiber-optic sensors to record internal particulate temperature kinetics during thermal-UV-C processing and developing dynamic-bed processing systems. Furthermore, complementary investigations are required to evaluate the long-term lipid photo-oxidation of the grain surface under intensive UV-C exposure and to validate shelf-life stability. Future work should include a more comprehensive characterization of the naturally occurring microbiota, encompassing not only the molecular identification of surviving heat-resistant spore-forming bacteria but also the profiling of initial vegetative bacterial populations to provide a complete microbiological risk assessment. Overall, this combined treatment presents a viable, mild thermal processing alternative to conventional thermal processing, offering food producers a practical approach to enhancing microbial safety.

## Figures and Tables

**Table 1:** Independent variables and levels of the Box–Behnken design.

Factor	Independent Variable	−1	0	+1
A	Oven temperature (°C)	60	80	100
B	Thermal treatment time (min)	2	6	10
C	UV-C Dose (mJ/cm^2^)	214.8	644.4	1074.0

**Table 2:** Box–Behnken experimental matrix and observed response values for coarsely milled barley decontamination.

Run	Oven Temperature (A, °C)	Thermal Treatment Time (B, min)	UV-C Dose (C, mJ/cm^2^)	Mean Log Reduction	Mean a_w_	Mean ΔE
1	60 (−1)	2 (−1)	644.4 (0)	0.468 ^m^ ± 0.068	0.36 ^a^ ± 0.008	3.66 ^j^ ± 0.068
2	100 (+1)	2 (−1)	644.4 (0)	1.099 ^c^ ± 0.074	0.35 ^ab^ ± 0.001	4.96 ^g^ ± 0.071
3	60 (−1)	10 (+1)	644.4 (0)	0.652 ^k^ ± 0.073	0.34 ^b^ ± 0.005	9.82 ^a^ ± 0.083
4	100 (+1)	10 (+1)	644.4 (0)	1.532 ^a^ ± 0.056	0.35 ^ab^ ± 0.002	5.35 ^f^ ± 0.069
5	60 (−1)	6 (0)	214.8 (−1)	0.845 ^f^ ± 0.068	0.35 ^ab^ ± 0.009	3.17 ^k^ ± 0.068
6	100 (+1)	6 (0)	214.8 (−1)	0.786 ^g^ ± 0.077	0.35 ^ab^ ± 0.007	8.38 ^c^ ± 0.070
7	60 (−1)	6 (0)	1074 (+1)	0.695 ^j^ ± 0.074	0.34 ^b^ ± 0.002	5.68 ^e^ ± 0.075
8	100 (+1)	6 (0)	1074 (+1)	1.076 ^d^ ± 0.076	0.35 ^ab^ ± 0.001	3.09 ^l^ ± 0.076
9	80 (0)	2 (−1)	214.8 (−1)	0.704 ^i^ ± 0.077	0.35 ^ab^ ± 0.009	8.79 ^b^ ± 0.066
10	80 (0)	10 (+1)	214.8 (−1)	1.121 ^b^ ± 0.073	0.35 ^ab^ ± 0.007	4.81 ^h^ ± 0.075
11	80 (0)	2 (−1)	1074 (+1)	0.525 ^l^ ± 0.073	0.34 ^b^ ± 0.004	5.99 ^d^ ± 0.068
12	80 (0)	10 (+1)	1074 (+1)	0.996 ^e^ ± 0.074	0.34 ^b^ ± 0.005	3.83 ^i^ ± 0.067
13 (Center point)	80 (0)	6 (0)	644.4 (0)	0.743 ^h^ ± 0.069	0.34 ^b^ ± 0.006	3.17 ^k^ ± 0.047
14 (Center point)	80 (0)	6 (0)	644.4 (0)	0.747 ^h^ ± 0.074	0.34 ^b^ ± 0.005	3.19 ^k^ ± 0.044
15 (Center point)	80 (0)	6 (0)	644.4 (0)	0.749 ^h^ ± 0.073	0.34 ^b^ ± 0.005	3.15 ^k^ ± 0.021

Values are presented as mean ± SD (*n = 3*). Means followed by different superscript letters in the same column indicate statistically significant differences (Duncan’s test, *p* < 0.05).

**Table 3:** ANOVA *p*-values and statistical performance metrics for response.

Effect	Microbial Reduction Y1	Water Activity Y2	Total Color Difference Y3
A: Oven Temperature	0.0497 *	0.4032 ^ns^	0.9346 ^ns^
B: Thermal Treatment Time	0.0486 *	0.1275 ^ns^	0.9503 ^ns^
C: UV-C Dose	0.7888 ^ns^	0.0409 *	0.3420 ^ns^
AB	0.5704 ^ns^	0.0493 *	0.2499 ^ns^
AC	0.3325 ^ns^	0.2532 ^ns^	0.1384 ^ns^
BC	0.9004 ^ns^	1 ^ns^	0.6996 ^ns^
AA	0.3672 ^ns^	0.0268 *	0.4234 ^ns^
BB	0.4292 ^ns^	0.1219 ^ns^	0.1837 ^ns^
CC	0.9642 ^ns^	0.562 ^ns^	0.4624 ^ns^

ns: Non-significant (*p* > 0.05); * Significant (0.01 < *p* ≤ 0.05).

**Table 4:** Model equations and statistical parameters for Y1, Y2, and Y3.

Response Variables	Model Equations	Model *p*-Value	Lack-of-Fit *p*-Value	Model F-Value	R^2^	Adjusted R^2^	SD	C.V (%)
Logarithmic Reduction (Y1)	Y1= 2.54715 − 0.0437917 A − 0.089125 B − 0.00115427 C + 0.000264479 AA + 0.00573698 BB + 2.73178E-8 CC + 0.000778125 AB + 0.0000128026 AC + 0.00000785615 BC	0.0448 *	0.0002 ^†^	3.95	79.927	60.70	0.0173	14.5
Water Activity a_w_ (Y2)	Y2 = 0.500625 − 0.003 A − 0.0084375 B − 0.0000407356 C + 0.000015625 AA + 0.000234375 BB + 6.77301E-9 CC + 0.0000625 AB + 2.90968E-7 AC	0.0351 *	0.5400 ^ns^	4.85	86.607	68.75	0.003	1.02
Total Color Difference ΔE (Y3)	Y3= 7.3912 − 0.15062 A − 0.049062 B + 0.00826641 C + 0.00250938 AA + 0.111016 BB + 0.00000496461 CC − 0.018 AB − 0.000226955 AC + 0.000263326 BC	0.5084 ^ns^	0.0000 ^†^	1.05	65.320	43.07	2.2134	27.08

ns: non-significant lack-of-fit (*p* > 0.05); * Statistically significant model (*p* ≤ 0.05); †: Significant lack-of-fit (*p* ≤ 0.05) attributable to the near-zero pure error variance across center-point replicates (high experimental repeatability) rather than poor model adequacy. R^2^, Adjusted R^2^ and C.V. (coefficient of variation) are expressed in %.

**Table 5:** Multi-response desirability optimization conditions, model predictions, and experimental validation results.

Response	Objective	Predicted Values	Actual Experimental Values	RSE (%)
Y1: Logarithmic Reduction	Maximize	1.198	1.385 ± 0.013	15.6
Y2: Water Activity a_w_	Minimize	0.34	0.34 ± 0	0
Y3: Total Color Difference ΔE	Minimize	2.60	2.77 ± 0.08	6.53

Y1 expressed as Log CFU/g.

## Data Availability

The data that support the findings of this study are available from the corresponding author upon reasonable request.

## Supplementary Materials

The following supporting information can be downloaded at https://www.mdpi.com/article/10.3390/foods15213962/s1, Figure S1: Diagnostic residual plots for the Logarithmic Reduction (Y1) response surface model. (a) Normal probability plot of standardized residuals, (b) Plot of residuals versus predicted values. Figure S2: Diagnostic residual plots for the water activity (Y2) response surface model. (a) Normal probability plot of standardized residuals, (b) Plot of residuals versus predicted values. Figure S3: Diagnostic residual plots for the total color difference (Y3) response surface model. (a) Normal probability plot of standardized residuals, (b) Plot of residuals versus predicted values. Table S1. Individual optimal values and experimental conditions of responses Y1, Y2 and Y3.

## Abbreviations

The following abbreviations are used in this manuscript:

UV-C	Ultraviolet C
RSE	Relative standard error
a_w_	Water activity
ΔE	Total color difference
RSM	Response surface modeling
PEF	Pulsed electric field
CP	Cold plasma
Kg	Kilogram
BBD	Box–Behnken Design
min	Minute
°C	Degree Celsius
mJ/cm^2^	millijoules per square centimeter
AA	Quadratic term for Factor A
BB	Quadratic term for Factor B
CC	Quadratic term for Factor C
AB	Interaction between Factor A and Factor B
AC	Interaction between Factor A and Factor C
BC	Interaction between Factor B and Factor C
A	Oven temperature
B	Thermal treatment time
C	UV-C dose
