# Supplementary material for: Multi-Response Optimization of Sequential Mild Heating and UV-C Radiation Hurdle Technology for Decontamination and Quality Retention of Traditional Tunisian Coarsely Milled Barley (Chorba Chiir)
Source: Foods. 2026 Nov 4;15(21):3962. doi: 10.3390/foods15213962 (PMC13762977; doi:10.3390/foods15213962)
Supplement: Supplementary file 1 [file foods-15-03962-s001.zip › foods-4595640-supplementary.pdf]

VResponse Surface Modeling and Multi-Response Optimization of Sequential Mild Heating and UV-C Radiation Hurdle Technology for Decontamination and Quality Retention of Traditional Tunisian Coarsely Milled Barley (Chorba Chiir).

Eya BenSalem [1, 2,*], Taroub Bouzaiene [3], Rania Zayani [1], Mohamed Boussaid [1], Carole Chaix [2], Chokri Messaoud[1]

Supplementary material

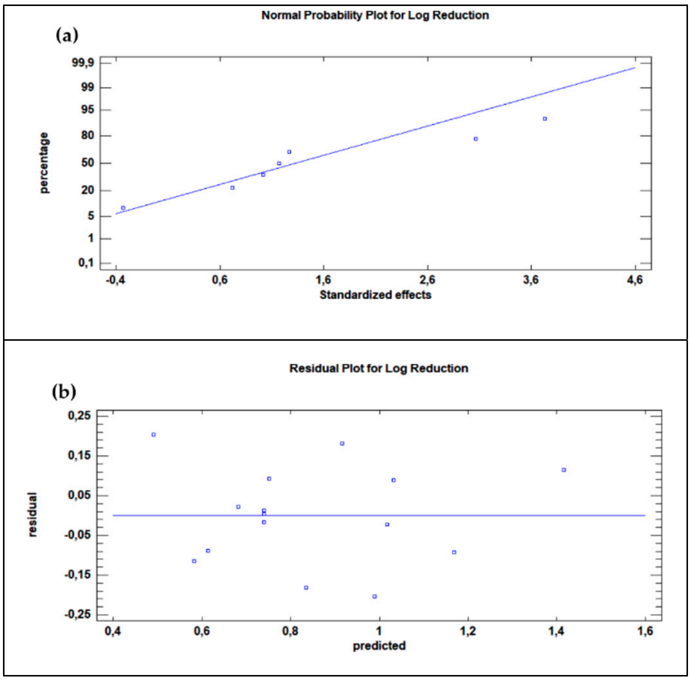

Figure S1: Diagnostic residual plots for the Logarithmic Reduction (Y1) response surface model. (a) Normal probability plot of standardized residuals, (b) Plot of residuals versus predicted values.

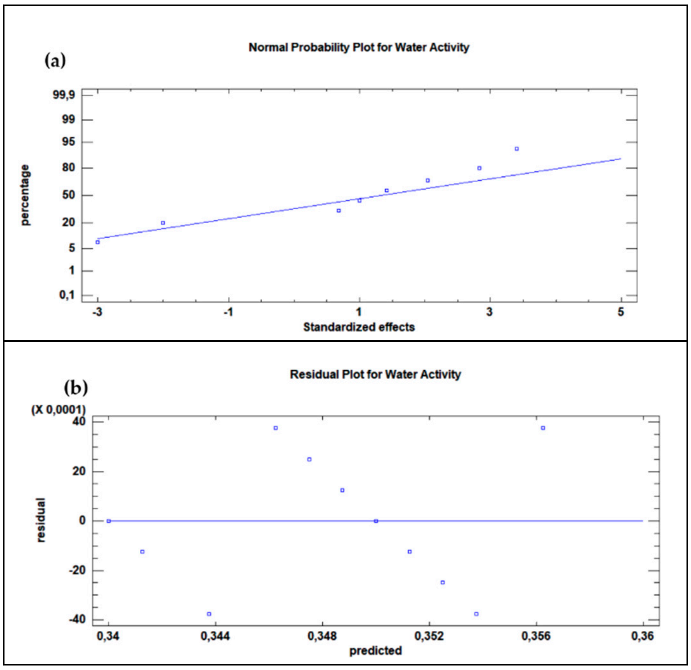

Figure S2: Diagnostic residual plots for the Water activity (Y2) response surface model. (a) Normal probability plot of standardized residuals, (b) Plot of residuals versus predicted values.

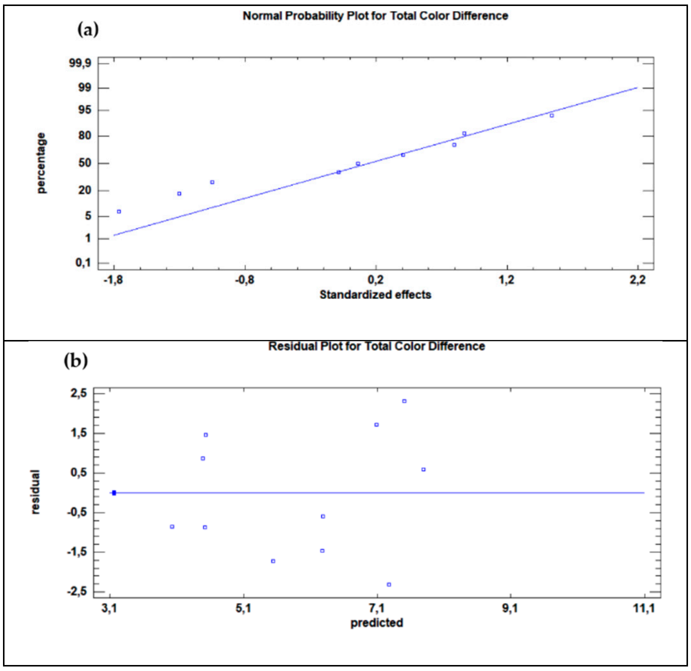

Figure S3: Diagnostic residual plots for the Total Color Difference (Y3) response surface model. (a) Normal probability plot of standardized residuals, (b) Plot of residuals versus predicted values.

Note: (a) Normal probability plot of standardized residuals, demonstrating that experimental errors closely follow the linear reference line (normality assumption). (b) Plot of residuals versus predicted values, showing a random, unstructured distribution around the zero baseline, confirming model linearity and constant variance (homoscedasticity).

Table S1. Individual optimal values and experimental conditions of responses Y1, Y2 and Y3.

**Response variables**	**Objective**	**Model equations**	**Optimal value**	**Optimal experimental conditions**
Logarithmic Reduction (Y1)	Maximize	Y1= 2.54715 - 0.0437917 A - 0.089125 B - 0.00115427 C + 0.000264479 AA + 0.00573698 BB + 2.73178E-8 CC + 0.000778125 AB + 0.0000128026 AC + 0.00000785615 BC	1.52 Log CFU/g	A= 100 °C<br>B= 9.99 min<br>C=1074 mJ/cm²
Water Activity a_w (Y2)	Minimize	Y2 = 0.500625 - 0.003 A - 0.0084375 B - 0.0000407356 C + 0.000015625 AA + 0.000234375 BB + 6.77301E-9 CC + 0.0000625 AB + 2.90968E-7 AC	0.33	A= 68.1 °C<br>B= 8.9 min<br>C=1074 mJ/cm²
Total Color Difference **ΔE** **(**Y3)	Minimize	Y3= 7.39125 - 0.150625 A - 0.0490625 B + 0.00826641 C + 0.00250938 AA + 0.111016 BB + 0.00000496461 CC - 0.018 AB - 0.000226955 AC + 0.000263326 BC	2.12	A= 100 °C<br>B= 7.05 min<br>C=1074 mJ/cm²
